# Scoping review protocol of prehabilitation interventions for primary arthroplasty

**DOI:** 10.4102/sajp.v79i1.1831

**Published:** 2023-03-22

**Authors:** Prithi Pillay-Jayaraman, Stacy Maddocks, Verusia Chetty

**Affiliations:** 1 Chris Hani Baragwanath Academic Hospital, Gauteng Department of Health, Johannesburg, South Africa; 2 Department of Physiotherapy, Faculty of Health Sciences, University of KwaZulu-Natal, Durban, South Africa; 3 Department of Physiotherapy, Faculty of Health Sciences, University of the Witwatersrand, Johannesburg, South Africa

**Keywords:** prehabilitation, arthroplasty, exercise, rehabilitation, scoping review, pre-operative optimisation

## Abstract

**Background:**

Osteoarthritis (OA) ranks fifth among all forms of disability worldwide and primary replacement arthroplasty is the treatment of choice in late-stage OA. The current situation in South Africa is that the waiting lists for arthroplasty are extensive with steep costs. According to many studies, physiotherapists can have an impact on this situation by implementing prehabilitation.

**Objectives:**

The aim of our study is to identify the trends in the literature regarding the content of prehabilitation programmes as well as the gaps.

**Method:**

The methodology will involve a literature search and the methodology as proposed by the Joanna Briggs Institute guidelines. The literature searches will be conducted in electronic databases and peer-reviewed journal studies will be included based on predetermined inclusion criteria. Two reviewers will screen all citations and full-text articles and the first author will abstract the data.

**Results:**

The results will be organised into themes and sub-themes, summarised, and reported as a narrative synthesis.

**Conclusion:**

The proposed scoping review will map the breadth of knowledge available on the topic of prehabilitation in terms of exercise prescription principles, pre-operative optimisation and gaps.

**Clinical implications:**

This scoping review is the first part of a study that aims to design a prehabilitation programme suitable for the South African public health user as the demographic and physical characteristics of its health users are unique and dependent on the context.

## Introduction

Osteoarthritis ranks fifth among all forms of disability worldwide (Cisternas et al. 2016) and it is estimated that 30.8 million adults have osteoarthritis with musculoskeletal disorders representing a global threat to healthy ageing (Murray et al. 2012). Most of the prevalence data on arthritis have been collected in studies conducted in developed countries. However, data from the 2010 global burden of disease study provide some evidence that lower- and middle-income countries (LMICs) such as India and South Africa may have greater arthritis prevalence than high-income countries. Data report that LMICs have 90% of the global burden of disease but only 12% of global health spending (Brennan-Olsen et al. 2017). In South Africa, a meta-analysis carried out on prevalence showed there was 55.1% prevalence of osteoarthritis in urban settings and the incidence ranges between 29.5% and 82.7% in adults over 65 years of age in rural settings. The study was carried out in 2015 and since then there have been no further studies or data available on the topic (Usenbo et al. 2015).

When conservative management of rheumatoid arthritis (RA) and osteoarthritis (OA) fails and the overall quality of life continues to decline for an individual, primary replacement arthroplasty is the surgical treatment of choice to alleviate joint destruction, decrease pain and improve quality of life. Arthroplasty is also recommended as a successful treatment option for late-stage OA (Dunn 2012). Replacement arthroplasties have excellent functional results and total hip replacement has been referred to as the ‘operation of the century’ as more than 95% of patients have been entirely satisfied with the functional results (De l’Escalopier, Anract & Biau 2016). High patient satisfaction is because of the marked decrease in pain and improvements in function with patients being able to participate in activities of daily living that they were previously unable to do within approximately 2 months of the operation (De l’Escalopier et al. 2016).

The current situation in South Africa, which is an LMIC, is that the waiting lists for arthroplasty are extensive with steep costs involved in the management of these patients in public sector hospitals that serve most of the population (Abera Abaerei, Ncayiyana & Levin 2017; Kavalieratos, Nortje & Dunn 2017). This long waiting period can be attributed to discrepancies between available resources, costs of the replacement arthroplasty and the necessary prioritisation of trauma-related procedures leaving very few beds and operating theatres for elective surgeries. This results in patients having to wait for years for arthroplasty procedures, which is a situation unique not only to South Africa (Dunn 2012; Kavalieratos et al. 2017).

In light of the above information, one of the ways in which a physiotherapist can impact the situation of high costs and long waiting times is to implement measures to reduce the length of hospital stay postoperatively (Masaracchio et al. 2017). Length of stay can be impacted by intensive physiotherapy sessions post-operatively and this will directly assist with early discharge creating more beds in the unit for these patients and therefore the waiting times for arthroplasty (Masaracchio et al. 2017). Literature searches around the topic revealed that most studies stipulate bi-daily physiotherapy to assist with early discharge (Masaracchio et al. 2017).

Another measure that can go hand in hand with the above is to adequately prepare, educate and support patients while waiting for the surgery by physical and psychosocial measures with a contextually relevant prehabilitation programme. A quick literature search on the topic of physiotherapy in reducing waiting lists and decreasing length of stay shows that prehabilitation as well as pre-operative education have a significant impact on improving outcomes (Clode, Perry & Wulff 2018; Gill & McBurney 2013; McKay, Prapavessis & Doherty 2012; Saw et al. 2016; Swank et al. 2011; Wallis & Taylor 2011). A meta-analysis of 49 published and unpublished studies shows that the average hospital stay is reduced by 12% and there is a mean reduction of 1.25 days when pre-operative education is incorporated as a part of the package of care (Wallis & Taylor 2011). A study by Crowe and Henderson also report that a prehabilitation programme of exercises and education has a direct impact in reducing the length of stay (Crowe & Henderson 2003).

There is only one South African-based study on prehabilitation carried out by Saw et al. in 2016. The intervention included six physiotherapist-led group-based sessions that included 2 h a week of education, exercise and relaxation at two public hospitals in South Africa. This is one of the few studies carried out by physiotherapists that incorporated patient education as part of prehabilitation and the components of education included education about the condition, self-management of pain and other symptoms, stress management and lifestyle education. Clode et al. (2018) conclude that group education talks about what to expect benefit patients and have a direct bearing on positive outcomes that are also statistically significant.

The types of exercises included were resistance training using resistance bands or weights, flexibility training and functional training such as step training. The studies that described the regime, followed the routine of warm-up exercises followed by resistance training exercises and step training ending with cool-down exercises and static stretching. Most studies indicated that patients come for therapy three times a week, 4–8 weeks prior to the operation (Clode et al. 2018; Gill & McBurney 2013; McKay et al. 2012; Saw et al. 2016; Swank et al. 2011; Wallis & Taylor 2011).

This preliminary review of the literature has in broad terms identified the trends in evidence regarding the content and type of exercises to include in a prehabilitation programme as well as the areas that are traditionally not included as a part of the prehabilitation programme. This provides a justification for conducting a scoping review. The scoping review will allow for a thorough, systematic and in-depth review of the literature to inform the first author on the topic at hand and allow for all relevant literature to be exhaustively analysed and critiqued and described prior to constructing the tenets of the prehabilitation programme that will be contextually relevant to a resource-constrained health system. Therefore, the objective of this scoping review is to identify and map newly reported prehabilitation interventions for patients with arthroplasty (between 1995 and 2020). It is anticipated that the results of our study will provide consensus on the identification and mapping of key aspects of care to include in a prehabilitation exercise programme prior to arthroplasty. Our study will attempt to locate and report on all available studies that have examined prehabilitation in the above population as well as the gaps in this area.

## Methodology

Our protocol was registered within the Open Science Framework platform (registration ID of this study protocol was reported in accordance with the reporting guidance provided in the Preferred Reporting Items for Systematic Reviews and Meta-Analyses protocols (PRISMA-P) statement and the PRISMA extension for scoping reviews (PRISMA-ScR; Appendix 1).

The methodology will involve conducting a scoping review to map, explore and study the breadth of information available on the topic of prehabilitation and identify gaps in the literature. A scoping review methodology is best suited as it allows for a rapid review of a comprehensive range of literature that includes all levels of evidence which can then be described in a detailed manner. It, however, excludes opinion articles and commentaries. The scoping review strategy as described by Arksey and O’Malley (2005) detailing five stages will be used. The five steps in the following sequence guided the manner in which the scoping review will be conducted: (1) defining the research question, (2) identifying the relevant studies, (3) selecting the main theme for our study, (4) charting and collecting the data and (5) summarising and reporting the results (Arksey & O’Malley 2005).

### Defining and pinpointing the research question

The main research question for the scoping purpose is, ‘What are the prehabilitation interventions for patients undergoing Primary Joint Arthroplasty?’

The areas of consideration and the sub-questions under the umbrella research question include the following:

- What are the types of exercises included in prehabilitation programmes for patients awaiting Primary Joint Arthroplasty?
- What is the intensity, frequency and duration of prehabilitation exercises?
- Does the prehabilitation programme cover aspects of psychosocial rehabilitation and if yes what is the content?
- Do the studies consider the impact of concurrent joint pain, deformities and their contribution to post-operative recovery?

### Information sources and search strategy

Identification of studies relevant to our review will be achieved through the utilisation of the search strategy as recommended by Peter et al. (2015). In the search for studies, computer databases such as Google Scholar, CINAHL, MEDLINE, PubMed, EBSCO host and Cochrane Library will be used. Any studies carried out between 1995 and 2020 will be included in the search strategy. The Boolean terms ‘and’; ‘or’; ‘not’ will be used to separate keywords. Additional potentially relevant studies will be identified by conducting a search of the references of the included articles, and further searches on websites such as the World Health Organization (WHO) and the Directory of Arthroplasty. Relevant grey literature will be identified through targeted searches of theses on ProQuest Dissertations and Theses Global, and conference abstracts on EMBASE Conference Abstracts and Conference Proceedings Citation Index-Science, Social Science and Humanities. Search terms will include knee, hip, joint replacement, arthroplasty, physiotherapy, physical therapy, exercise, rehabilitation, prehabilitation, pre-operative, level of knowledge, patient education, foot deformities, osteoarthritis and patient compliance. Inclusion criteria for the scoping review will include exercise or education about prehabilitation and studies will be selected based on how appropriate they are to our study question however ensuring that the selection process is iterative and inclusive of grey literature and diverse study designs (O’Brien et al., 2016)

### Selection of eligible studies

The population concept context (PCC) framework (Table 1) will guide the process of study selection and its link with the research question. To be included in the review, studies need to have a sample population of adults, 45 years and older as this is the common age for arthroplasty; whose participants have had a lower limb joint replacement or are scheduled to have one; measure or focus on functional outcomes pre- and post-arthroplasty; interventions and exercise that incorporate pre-operative optimisation which may or may not include education as a preparation for joint arthroplasty as is contained in the proposed conceptual framework. Our study will elaborate on the exercise prescription and regime used as a part of the intervention, for example, describing the type of exercises prescribed, such as, strengthening, flexibility, resistance, balance and functional activities and if any educational interventions are incorporated. The studies will also be scrutinised for the principles of exercise prescription used in terms of repetitions and frequency of interventions and their correlation to efficacy if described. Peer-reviewed journal articles will be included if they are written in English, involve human participants and describe measures for physical, psychological and functional status of patients, and contain physical activity/exercise and/or the recommendations prior to arthroplasty and post-arthroplasty. Quantitative (e.g. randomised controlled trials, systematic reviews, observational studies, cohort and case-control), qualitative and mixed-method studies will be included if they consider the above aspects as the purpose of the study. Studies will be excluded if aims and the study population do not fit into the conceptual framework of our study or do not include any aspect of pre-operative optimisation.

**TABLE 1:** Population concept context framework for eligibility of studies.

Criteria	Determinants
P-Population	Adults 45 years and older living with osteoarthritis
C-Concept	Physical activity, exercise rehabilitation, aerobic, anaerobic, strengthening, flexibility, resistance and balance exercises; assistive device usage, education on operation, education of rehabilitation, prehabilitation and pre-operative optimisation
C-Context	Quality of life, functional outcomes, activities of daily living and outcome measures

### Inclusion criteria

All articles or studies eligible for selection will meet the following inclusion criteria:

- Articles that include prehabilitation in arthroplasty patients
- All published peer-reviewed research articles
- Articles written in English.

### Exclusion criteria

Articles or studies will be excluded if they have any of the following criteria:

- Studies where full-text articles cannot be obtained
- Commentaries or opinion pieces.

Eligible articles will be uploaded into endnote software for Windows 10 to ensure the identification and removal of all duplicated articles. Three reviewers who are familiar with the study proposal will be involved in the scoping review process. Title, abstract and keywords screening of all eligible articles will be conducted by the first author (P.P.-J.) and second reviewer (H.E.). The process will entail that the two reviewers (P.P.-J. and H.E.) initially screen the citations by title, abstract and keywords to ensure that the selected studies fall within the paradigm of the conceptual framework. Excluded citations will be reviewed and confirmed by a third reviewer (V.C.). The next step will consist of obtaining full texts of all selected articles by undertaking a thorough and exhaustive search of the web. In those instances where the full text cannot be obtained from the web, a concerted effort will be made to obtain these full-text articles by engaging with the university subject librarian and or contacting the author(s) as necessary. Full-text screening as to whether the selected articles meet the inclusion criteria will be conducted by both reviewers independently (P.P.-J. and H.E.). Major discrepancies and a lack of agreement in the inclusion of the scoping review between both reviewers will be resolved through discussion. However, if there was still no resolution, a third reviewer (V.C.) will be employed to ensure consensus.

### Charting the data

**FIGURE 1:**
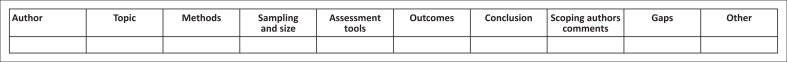
Charting data. Note: Collation, summarisation, and reporting of the results.

A standardised data charting table as depicted in Figure 1 will be used to categorise and summarise the extracted information. The tool will capture the relevant information on study design and other detailed information on the metrics used to describe pre-operative optimisation in arthroplasty patients. Information of interest will include the following:

- Demographic study characteristics: Authors, year of publication, journal, topic, setting and country of origin
- Study characteristics: Study design, aim/objective of the study, sampling strategy and sample size
- Participant characteristics: Population, sample, age (e.g. mean with standard deviation, range) and gender (e.g. percentage of male/female participants)
- Assessment tools: Anthropometric data, outcome measures used and intervals of assessment
- Interventions: Exercise prescription (e.g. type and duration or intensity), types of exercises, education component, intervals of exercises and model of delivery
- Outcome results (e.g. findings relevant to study objectives)
- Key relevant findings and conclusions
- Other fields to capture data relevant to the assessment of study validity
- Scoping review authors’ analysis.

The data-charting form will be jointly updated by the two reviewers to capture all permutations and combinations possible of the research question and to determine which variables to extract, for example, study design, population, sample characteristics, et cetera. The two reviewers will independently chart the data and any disagreements will be resolved through discussion between the two reviewers or further adjudication by a third reviewer.

The main research question is the primary factor guiding data collection and extraction; however, due consideration will also be given to studies that address the sub-questions. The data will be presented in a narrative format with the main categories describing the summaries of search results, study characteristics, exercise and psychosocial intervention. Information on the type of exercises, exercise prescription strategies, muscles targeted, and duration of intervention, etc. will be described in detail and the trends, similarities and gaps will be inferred and mapped. The psycho-social intervention subcategory explores the contents of the education component of prehabilitation and looks at the topics included to enhance and equip the patient prior to the surgery. In summary, all the information will be synthesised and presented in a narrative format which will include numerical and thematic information on the various types of interventions used as well as the gaps that are identified.

### Quality appraisal

Prior to performing a comprehensive charting process, a trail of methods will be carried out to enhance the methodology of our scoping study as recommended by Daudt et al. (2013).

## Discussion

Our proposed scoping review maps the breadth of knowledge available on the topic of prehabilitation interventions in patients scheduled to undergo joint arthroplasty in terms of exercise prescription principles and other ways to implement pre-operative optimisation and the effects of this on specified outcomes. Our scoping review is the first part of a study that aims to design a prehabilitation programme suitable for a resource-constrained LMIC health care system wherein the demographic and physical characteristics of its health users are unique and dependent on the context. It allows the first author to thoroughly interrogate the available literature and determine the feasibility and applicability of the information available to the context of an LMIC health care system. It also allows for identification and pinpointing of areas that have not been considered thus far, based on the clinical presentation and contextual elements inherent to LMIC thereby allowing the creation of a programme that best suits the needs and challenges of these health care users. It is envisaged that once a suitable programme is developed, it can be used as a template for a basic standard of care that is incorporated as the package of service delivery for arthroplasty. Our scoping review also allows for the identification of future research needs that allow for streamlining of care and perhaps even put in early intervention measures to delay the need for arthroplasty reducing the burden of care. This highlights the potential for cost-cutting and reduced health care expenditure to the stakeholders, healthcare managers and policy makers.

## Conclusion

A potential limitation of the scoping review methodology is the fact that only English language studies will be considered.

## Appendix 1

**TABLE 1-A1:** Preferred Reporting Items for Systematic Reviews and Meta-Analyses extension for Scoping Reviews (PRISMA-ScR) Checklist.

Item	Prisma-ScR checklist item	Reported on page #
**Title**		
1. Title	Identify the report as a scoping review	Page 1
**Abstract**		
2. Structured summary	Provide a structured summary that includes (as applicable): background, objectives, eligibility criteria, sources of evidence, charting methods, results and conclusions that relate to the review questions and objectives	Page 2, 3
**Introduction**		
**3.** Rationale	Describe the rationale for the review in the context of what is already known. Explain why the review questions/objectives lend themselves to a scoping review approach	Page 3, 4
4. Objectives	Provide an explicit statement of the questions and objectives being addressed with reference to their key elements (e.g. population or participants, concepts and context) or other relevant key elements used to conceptualise the review questions and/or objectives	Page 5
**Methods**		
5. Protocol and registration	Indicate whether a review protocol exists; state if and where it can be accessed (e.g. a web address) and if available, provide registration information, including the registration number	Page 6 Systematic review registration: OSF Center for Open Science: https://osf.io/9fdsh/
6. Eligibility criteria	Specify characteristics of the sources of evidence used as eligibility criteria (e.g. years considered, language and publication status), and provide a rationale	Page 7
7. Information sources†	Describe all information sources in the search (e.g. databases with dates of coverage and contact with authors to identify additional sources), as well as the date the most recent search was executed	Page 7
8. Search	Present the full electronic search strategy for at least one database, including any limits used, such that it could be repeated	Information included and on Page 10
9. Selection of sources of evidence‡	State the process for selecting sources of evidence (i.e. screening and eligibility) included in the scoping review	Included in the methodology To be carried out in the review
10. Data charting process§	Describe the methods of charting data from the included sources of evidence (e.g. calibrated forms or forms that have been tested by the team before their use, and whether data charting was done independently or in duplicate) and any processes for obtaining and confirming data from investigators	Data charting is included on Page 9,10
11. Data items	List and define all variables for which data were sought and any assumptions and simplifications made	N/A
12. Critical appraisal of individual sources of evidence¶	If done, provide a rationale for conducting a critical appraisal of included sources of evidence; describe the methods used and how this information was used in any data synthesis (if appropriate)	N/A
13. Synthesis of results	Describe the methods of handling and summarising the data that were charted	N/A at this stage but plan included in methodology
**Results**		
14. Selection of sources of evidence	Give numbers of sources of evidence screened, assessed for eligibility, and included in the review, with reasons for exclusions at each stage, ideally using a flow diagram	N/A at this stage but we will include in final write up
15. Characteristics of sources of evidence	For each source of evidence, present characteristics for which data were charted and provide the citations	Data chart has been provided in methodology but at this stage the final review is not complete
16. Critical appraisal within sources of evidence	If done, present data on critical appraisal of included sources of evidence (see item 12).	MMAT will be used as described in methodology on the final review
17. Results of individual sources of evidence	For each included source of evidence, present the relevant data that were charted that relate to the review questions and objectives	N/A at this stage
18. Synthesis of results	Summarise and/or present the charting results as they relate to the review questions and objectives	N/A at this stage
**Discussion**		
19. Summary of evidence	Summarise the main results (including an overview of concepts, themes and types of evidence available), link to the review questions and objectives, and consider the relevance to key groups	N/A at this stage
20. Limitations	Discuss the limitations of the scoping review process	N/A at this stage
21. Conclusions	Provide a general interpretation of the results with respect to the review questions and objectives, as well as potential implications and/or next steps	N/A at this stage
**Funding**		
22. Funding	Describe sources of funding for the included sources of evidence, as well as sources of funding for the scoping review. Describe the role of the funders of the scoping review	N/A at this stage

*Source:* Tricco, A.C., Lillie, E., Zarin, W., O’Brien, K.K., Colquhoun, H., Levac, D. et al., 2018, ‘PRISMA Extension for Scoping Reviews (PRISMA-ScR): Checklist and explanation’, *Annals of Internal Medicine* 169(7), 467–473. https://doi.org/10.7326/M18-0850

JBI, Joanna Briggs Institute; PRISMA-ScR, Preferred Reporting Items for Systematic reviews and Meta-Analyses extension for Scoping Reviews; MMAT, Mixed Methods Appraisal Tool; N/A, not applicable.

† , Where *sources of evidence* (see second footnote) are compiled from, such as bibliographic databases, social media platforms and websites;

‡ , A more inclusive/heterogeneous term used to account for the different types of evidence or data sources (e.g. quantitative and/or qualitative research, expert opinion and policy documents) that may be eligible in a scoping review as opposed to only studies. This is not to be confused with *information sources* (see first footnote);

§ , The frameworks by Arksey and O’Malley 2005 (6) and Levac et al. 2010 (7) and the Peter et al. 2015 (JBI guidance) (4, 5) refer to the process of data extraction in a scoping review as data charting;

¶ , The process of systematically examining research evidence to assess its validity, results and relevance before using it to inform a decision. This term is used for items 12 and 19 instead of ‘risk of bias’ (which is more applicable to systematic reviews of interventions) to include and acknowledge the various sources of evidence that may be used in a scoping review (e.g. quantitative and/or qualitative research, expert opinion and policy document).
